# The Impact of the Lung Ultrasound on the Diagnosis of Interstitial Pulmonary Edema Before Extubation After Gynecologic Laparoscopic Surgery in the Trendelenburg Position: A Case Report

**DOI:** 10.7759/cureus.74016

**Published:** 2024-11-19

**Authors:** Vasileios Boviatsis, Alexios Triantopoulos, Paraskevi Dedopoulou, Christos Georgakopoulos

**Affiliations:** 1 Anesthesiology, General Hospital of Patras, Patras, GRC; 2 Surgery, General Hospital of Patras, Patras, GRC

**Keywords:** acute kidney injury, diuretics, extravascular lung water, laparoscopy, lung, pregnancy, pulmonary edema, ultrasonography

## Abstract

Lung ultrasound contributes to the diagnosis of perioperative pulmonary edema due to fluid overload and impairment of renal function. Laparoscopic surgery and the patient’s intraoperative position can facilitate the emergence of these disturbances as well. A 34-year-old female patient underwent laparoscopic salpingectomy and ovarian resection in the Trendelenburg position because of an unruptured ectopic pregnancy. The large fluid volume administration pre- and intraoperatively and the intraoperative low urine output led to the development of interstitial pulmonary edema before extubation. The formation of comet-tail lines at the lung ultrasound indicated the administration of diuretic therapy, followed by the improvement of the patient’s cardiopulmonary status, urine excretion, and extubation. A subsequent chest X-ray verified the foregoing findings. Lung ultrasound enables the quick and accurate diagnosis of interstitial edema. The early administration of diuretic therapy eliminates the potential of the patient’s prolonged postoperative hospitalization, even in the intensive care unit.

## Introduction

An ectopic pregnancy, constituting a minority of all pregnancies, is estimated to occur in approximately 2% of cases [1]. The implantation of the zygote outside the uterine cavity is mostly located in the fallopian tube, which is assessed by diagnostic laparoscopy and is followed by the safe laparoscopic resection of the product of conception with its surrounding tissues when feasible [2]. The surgical pneumoperitoneum is a prerequisite for performing laparoscopic surgeries and is mainly induced by CO_2_ insufflation due to its greater patient tolerability compared to other gases [3]. However, the applied high intra-abdominal pressure and the Trendelenburg position not only enhance the development of hypercarbia and acidosis but also mediate the reduced renal perfusion and urine output [4]. The impaired renal function, along with fluid overload and laparoscopy, may lead to perioperative pulmonary edema, which can be diagnosed nowadays in the operating theatre with accuracy using lung ultrasound [5-7].

We report a case of interstitial pulmonary edema that developed in a patient with unruptured ectopic pregnancy during laparoscopic salpingectomy and ovarian resection in the Trendelenburg position, just before extubation. The complication was addressed early by sonographic imaging and managed appropriately. Informed written consent to publication was obtained from the patient during her postoperative hospitalization.

## Case presentation

A 34-year-old female patient, 63 kg body weight and with a height of 162 cm, was diagnosed with unruptured ectopic first-trimester pregnancy located at the right fallopian tube. Her medical history was negative for major illnesses and medication, drug and alcohol intake, previous surgeries, smoking, and known allergies. Except for abdominal and transvaginal ultrasound, no chest X-ray was conducted during the initial evaluation. The admission laboratory check showed a hematocrit of 34% and a hemoglobin level of 11 gr dl^-1^ without any other abnormal findings.

After receiving about 4 liters of crystalloid fluids (Ringer’s lactate solution) intravenously, the patient arrived at the operating theater around 20 hours after her admission. The preoperative vital signs included arterial pressure: 125/79 mmHg, heart rate: 76 beats per minute, and oxygen saturation via 100% SpO_2_. The subsequent patient’s induction to general anesthesia was smooth and was maintained with 1-1.5% of sevoflurane, 55% of N_2_O, and 45% of O_2_ with flow at 1 L/min. A 100 ml solution of 0.9% sodium chloride (N/S 0.9%) containing 400 mg lidocaine, 200 mg tramadol, 5 gr magnesium sulfate, and 200 mcg dexmedetomidine was administered at a rate of 15-20 ml/h to provide intraoperative analgesia. The plane of anesthesia was monitored through the bispectral index, while noninvasive blood pressure measurement every three to five minutes, continuous electrocardiography, SpO_2_, and end-tidal CO_2_ were incorporated into the monitoring equipment. Furthermore, the patient’s bladder was catheterized using a 14 Fr urinary catheter to empty the bladder preoperatively and monitor the urinary output, beginning with an initial urine volume of 100 ml.

After the CO_2_ insufflation of the peritoneal cavity was performed by the implementation of the Hasson technique, an intraabdominal pressure of around 13 mmHg was achieved. However, fluctuations, with a range between 10 and 19 mmHg, were documented intraoperatively. Following the completion of pneumoperitoneum, the vital signs remained stable with arterial pressure of 132/84 mmHg, heart rate of 85 beats per minute, end-tidal CO_2_ of 36 mmHg, and SpO2 of 98%, and the patient was set at a 20-degree Trendelenburg position with a small tilt to the left. A subsequent laparoscopic right salpingectomy and ovarian resection were accomplished uneventfully, with an estimated blood loss of less than 150 ml. The duration of the operation was approximately 90 minutes, and the patient received about 800 ml of Ringer’s lactate solution, maintaining her hemodynamic stability, as shown in Table 1. Once the required hemostasis was performed, the pneumoperitoneum was released, and the laparoscope was removed. The patient was set again at the 0-degree supine position preoperatively, and the administration of inhaled anesthetics and intravenous analgesic agents was stopped. As soon as the sutured incisions were covered with sterilized gauze, a 200 mg sugammadex was administered intravenously.

**Table 1:** Intraoperative parameters and their values. SAP: Systolic arterial pressure, DAP: Diastolic arterial pressure, max: maximum, min: minimum, bpm: beats per minute, BIS: Bispectral Index, EtCO_2_: End-tidal CO_2 _

INTRAOPERATIVE PARAMETERS	VALUES
SAP max.-min./DAP max.-min. (mmHg)	138-111/89-68
Heart Rate (bpm)	68-94
SpO_2_ (%)	98-100
BIS	43-58
EtCO_2_ (mmHg)	34-39

**Figure 1:**
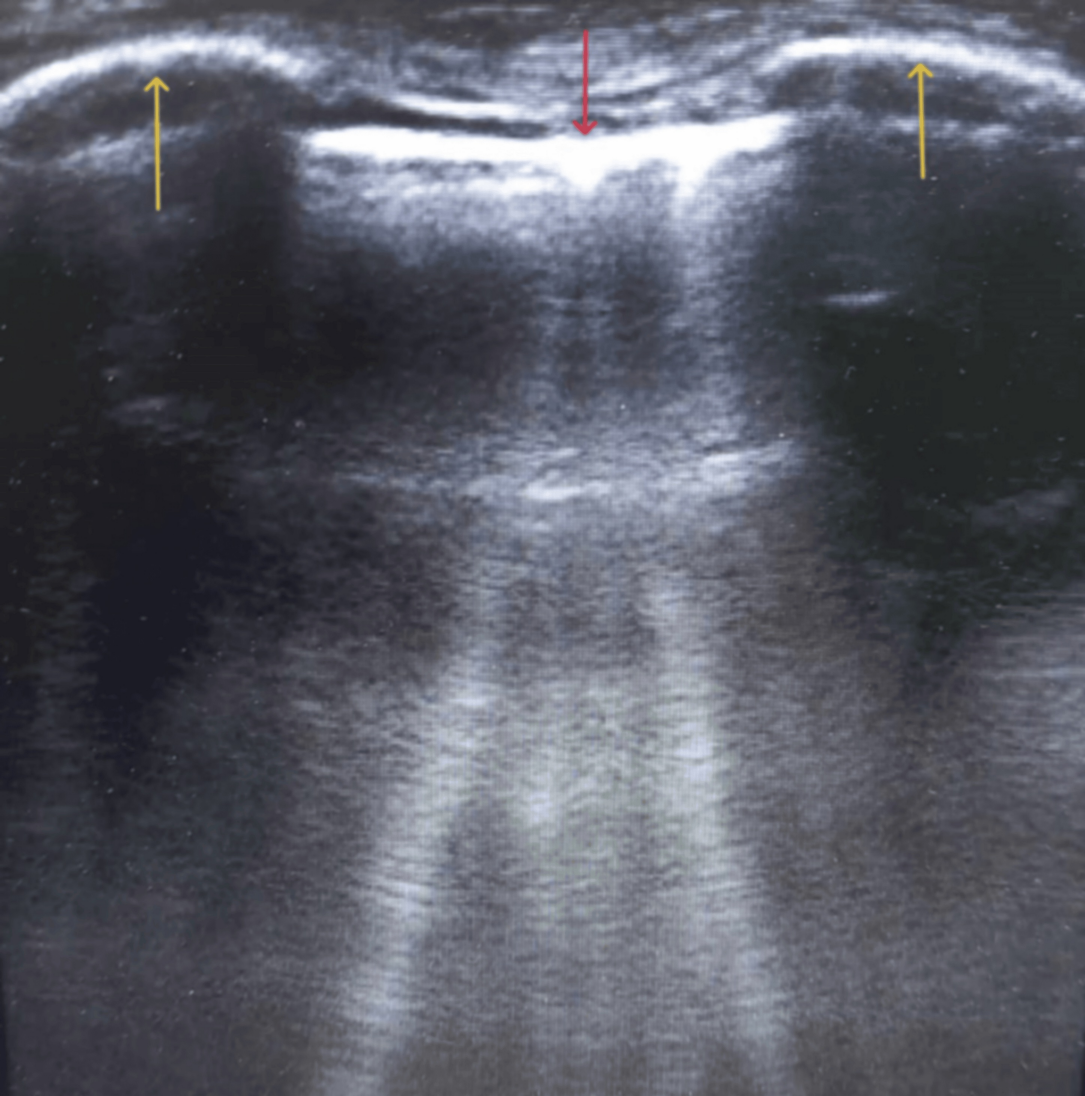
Right lung ultrasound image at the level of the parasternal line. The yellow arrows depict the fourth and fifth costal bones, while the red arrow shows the pleural line. The six coherent, hyperechogenic artifacts arising from the pleural line are defined as comet-tail or B lines.

**Figure 2:**
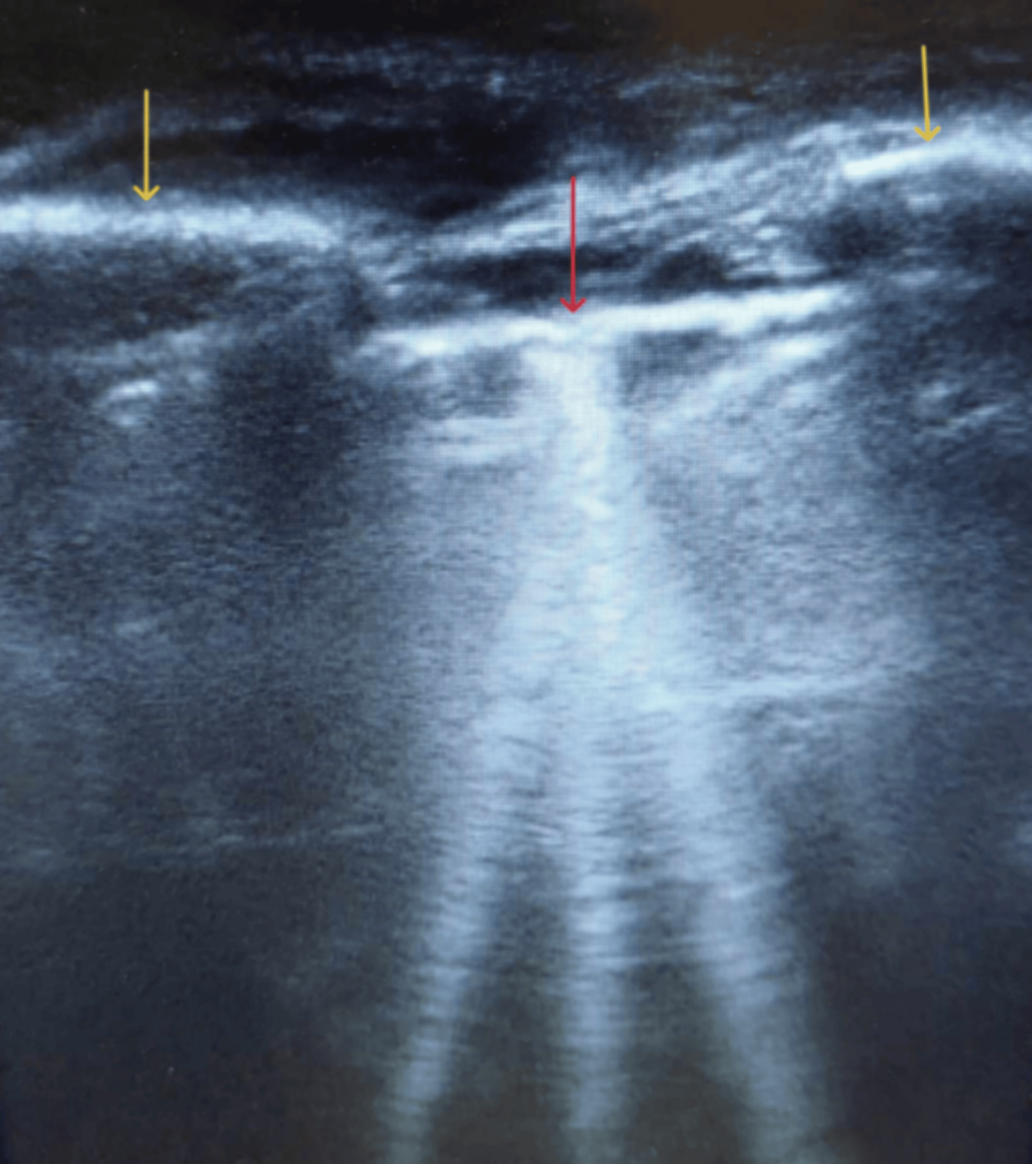
Right lung sonographic image at the level of the anterior axillary line. The yellow arrows show the third and fourth costal bones, and the red arrow demonstrates the pleural line. The depicted comet-tail lines are characterized by higher density than in Figure 1.

**Figure 3:**
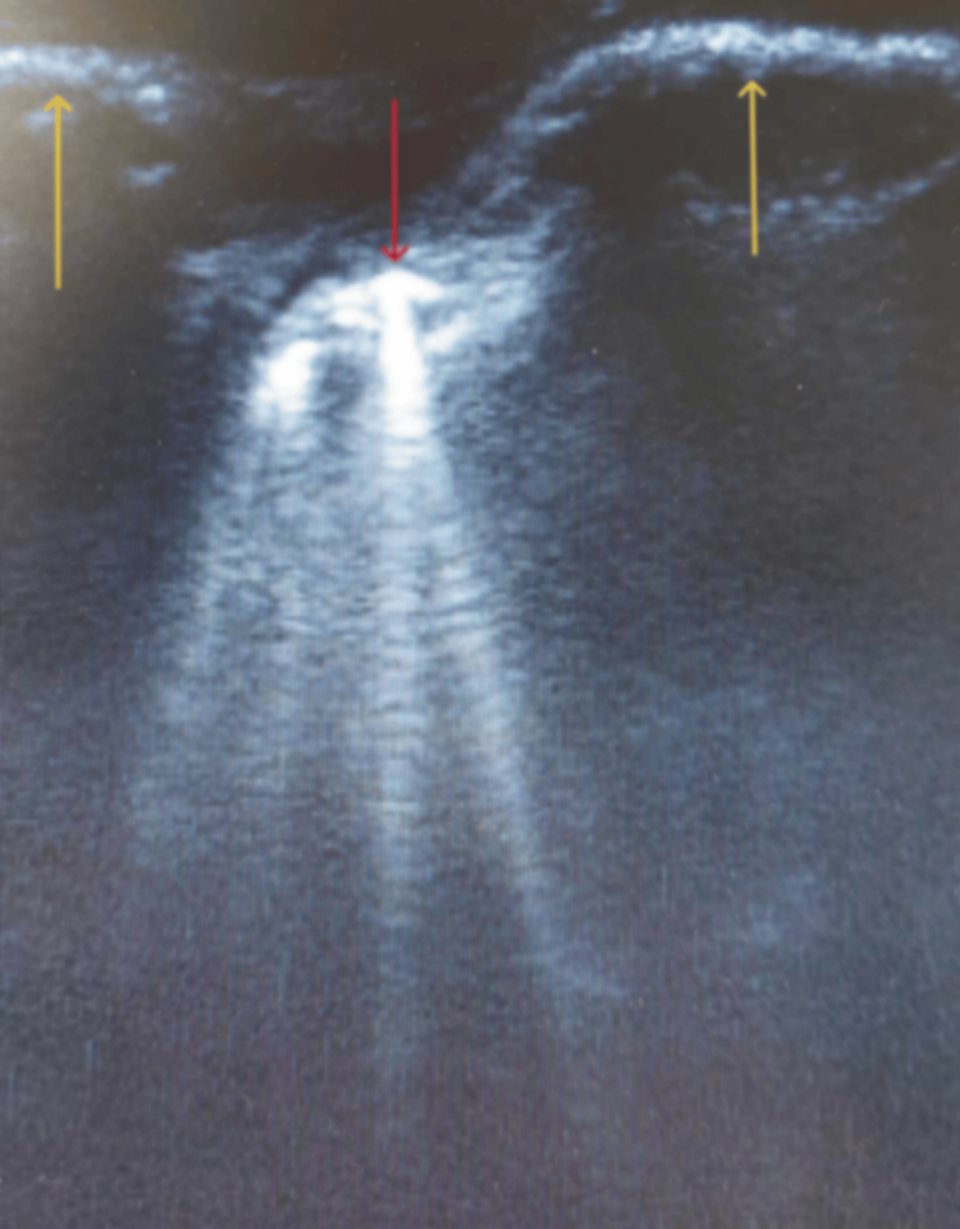
Left lung ultrasound image at the level of the parasternal line. Yellow arrows: fourth and fifth costal bones; red arrow: pleural line. The four well-defined hyperechoic artifacts are equivalent to comet-tail lines.

Despite the vital signs of the patient ranging within normal values (arterial pressure of 129/85 mmHg, heart rate of 89 bpm, SpO_2_ of 99%) and bispectral index ranging over 75, accompanied by the detection of a substantial increase in the electromyogram tone, the patient’s status began to deteriorate. Specifically, the rapid increase of the EtCO_2_ up to 48 mmHg, with a simultaneous gradual drop of SpO_2_ value at 90% and signs of respiratory distress, were observed, such as tachypnea (respiratory rate around 23 breaths per minute), low tidal volumes (< 6 ml kg^-1^), paradoxical chest and abdominal movements, intercostal muscle retractions, and tracheal tugging. Then 1 mg midazolam was administered IV for causing retrograde amnesia of the ongoing respiratory distress condition and for mild sedation, achieving a bispectral level of 72. The following auscultation revealed bilateral rhonchi and wheezes that were distributed across the anterior and lateral chest wall. The immediate ultrasound scanning of the anterior and lateral right and left chest wall depicted the <comet-tail artifacts>, which are associated with interstitial pulmonary edema (Figures 1-3).

**Figure 4:**
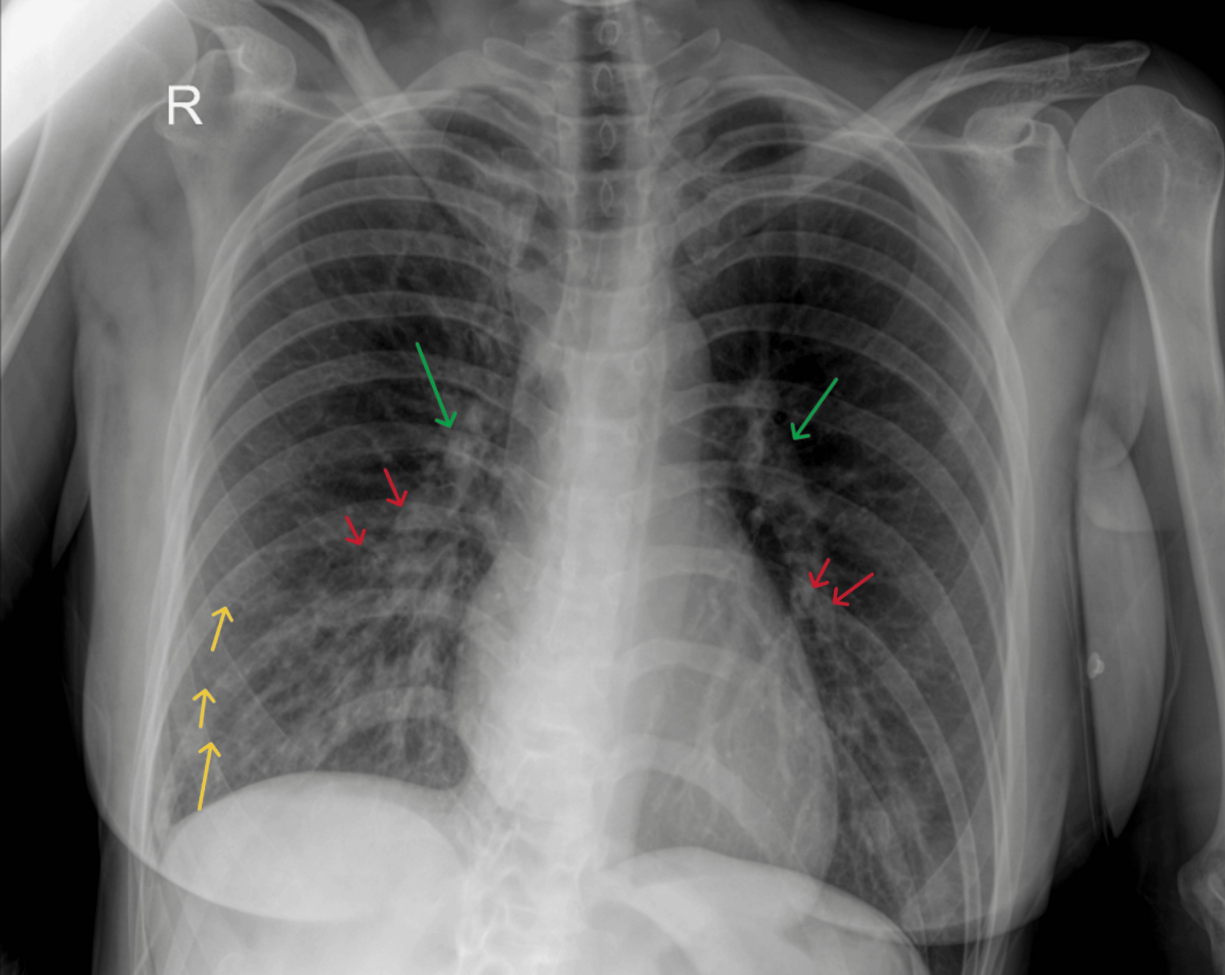
Chest X-ray. Kerley B lines (yellow arrows) are distinguished at the right lung periphery, along with peribronchial cuffing (red arrows), especially at the perihilar areas bilaterally. The diffuse enlargement of the peribronchovascular spaces (green arrows) is apparent bilaterally, especially at the level of the lung hilum.

This evidence, combined with the low urinary output of 50 ml from the beginning of the operation, led to the administration of 20 mg furosemide IV. The patient was safely extubated 20 minutes later after the urine output was increased to 1050 ml from the last measurement, the rapid optimization of the respiratory pattern was documented, including EtCO_2_ levels below 39 mmHg, a slower respiratory rate (< 20 breaths per minute), and higher tidal volumes (6-8 ml/kg), and a bispectral index value of 93 was achieved. No additional ultrasound imaging was executed prior to the extubation. The Glasgow Coma Scale score of the patient was 15/15, and the conducted clinical tests (5 seconds head lift, grip strength) for residual neuromuscular blockade were negative. Afterward, the patient was transferred to the post-anesthesia care unit, where a chest X-ray in a supine position (Figure 4) verified the diagnosis. The patient was discharged from the post-anesthesia care unit 1.5 hours later, having a stable cardiopulmonary status and a total urine output of 3 liters from the furosemide intravenous administration. 

## Discussion

The abnormal accumulation of fluid in the extravascular space of the lung due to the imbalance of the fluid drainage by the pulmonary vessel and lymphatic circulation is defined as pulmonary edema [7,8]. The increased hydrostatic pressure in the pulmonary capillaries enhances the fluid transudation in the interstitial compartment, leading to the emergence of interstitial edema [8]. Fluid overload and left-sided heart failure appear to be the dominant causes of this clinical entity [8].

In this case, the patient accidentally received a total fluid volume of 4.8 liters R/L intravenously in less than 24 hours, without including any extra preoperative oral and intravenous solution intake, while the patient’s daily fluid requirements were calculated at approximately 2.5-3 liters by commonly used formulas [9]. The patient did not require any blood transfusion, which may contribute to fluid overload, as the preoperative levels of hematocrit and hemoglobin were 34% and 11 gr dl^-1^, respectively, and the ectopic pregnancy was unruptured, with negligible intraoperative hemorrhage (< 150 mL) [7,10]. Also, the patient’s medical history was free of major cardiac disease, which was confirmed by echocardiography postoperatively, showing an ejection fraction over 60%, with no valvular disease, ventricle motion abnormalities, or dilation and pericardial effusion. Based on the above findings, the iatrogenic administration of the extra R/L volume of 2 liters contributed to the fluid overload and the interstitial edema development. Nevertheless, more aspects should be examined in depth.

The peritoneum insufflation was accomplished with the use of CO_2_, because of its high solubility and the low risk for gas embolism [4,11]. During laparoscopic surgeries, hypercarbia and subsequent acidosis are usually established, because of the high CO_2_ absorption from the peritoneal cavity into systematic circulation and the restricted CO_2_ elimination [11]. As a result, a centrally mediated sympathetic stimulation occurs, leading to tachycardia and systematic vasoconstriction in order to compensate for the decreased cardiac contractility and systematic vasodilation caused by hypercarbia [11]. The systematic vascular and pulmonary resistance, along with mean arterial pressure and central vein pressure, is further increased due to the sympathetic response triggered by the high intraabdominal pressure, especially > 15 mmHg, and the increased venous return induced by the Trendelenburg position [4,11,12]. These factors, in combination with the prolonged duration of the operation, amplify the hypercarbia as well, via the cephalad deviation of the diaphragm and its limited mobility [4,11]. These pathophysiologic mechanisms affected both the cardiac preload and afterload and contributed to the development of pulmonary edema in terms of the undergone surgery, even though there was no verification through invasive monitoring for central vein pressure, systemic vascular resistance, arterial, and pulmonary artery pressure. In addition, the high preoperative intravascular volume loading dose (> 10 ml/kg) was associated with the incidence of pulmonary edema too, which is compatible with the Gutt CN et al. study [11].

Another crucial factor that is related to pulmonary edema is the impaired renal function. The renal hypoperfusion and low glomerular filtration rate are mainly attributed to the high intraabdominal pressure (> 15 mmHg) that stimulates the corresponding pathophysiologic pathways [4,11]. During our laparoscopic surgery, the increased intraabdominal pressure aggravated the renal blood flow, leading to a transient prerenal acute renal failure and reduced urine output of 50 ml from the initial incision to the clinical assessment of the patient, just before extubation.

Once the symptoms and signs of the deterioration of the patient’s clinical status were detected, the lungs were evaluated via sonographic imaging. Lung ultrasound is a readily accessible and rapid imaging technique that provides noninvasively valuable insights into the lung parenchyma. This method is particularly useful when the implementation of the catheter-based thermodilution technique for the measurement of pulmonary wedge pressure, or the option to perform a chest X-ray, is not immediately available or feasible, as was the case in our situation [13]. A linear probe was utilized for the ultrasound examination, which was conducted on the anterior and lateral chest wall and was extended between the second and fifth intercostal spaces, ranging from parasternal to anterior axillary lines bilaterally [14]. The ultrasound imaging depicted the comet-tail lines, or B lines, distinct hyperechogenic artifacts originating from the pleural line and extending uniformly into the distal field of the ultrasound screen without fading [7,14]. Their diffusion was greater in the right lung compared to the left lung, and the zone of high density was located at the right anterior axillary line, third intercostal space (Figures 2, 3), which advocated the cardiogenic origin of the comet-tail lines [14]. 

The formation of the comet-tail lines relied on the reverberation phenomenon when the reflected sonographic beam reaches the thickened subpleural interlobular septa caused by the accumulated extravascular lung water [7,14]. This evidence, in combination with the display of 6 comet-tail lines (Figure 1), which is equivalent to pulmonary capillary wedge pressure of 12-18 mmHg, indicated the establishment of interstitial pulmonary edema, as lung ultrasound is associated with almost 100% sensitivity and specificity for acute pulmonary edema [7,14,15]. The administration of diuretic therapy was accompanied by the clinical improvement of the cardiopulmonary status, the urine excretion, and the following extubation of the patient. 

The foregoing ultrasound findings were confirmed by the conduction of chest X-ray (Figure 4) at the post-anesthesia care unit, although recent studies demonstrated the superiority of lung ultrasound over chest X-ray [7,14]. The Kerley B lines, the mild enlargement of the peribronchovascular spaces, and the apparent peribronchial cuffing in the perihilar areas were identified [7,8]. The derived data from the radiological imaging, along with the continuous increase of the urine output, ensured the validity of the initial diagnosis.

## Conclusions

During laparoscopic surgeries, any disturbance of the patient’s vital signs and respiratory pattern necessitates an early physical examination and clinical assessment of the patient. The subsequent lung ultrasound is a useful bedside modality that enables the accurate detection of acute lung abnormalities, including interstitial pulmonary edema, in the operating theater. Therefore, the early diagnosis, followed by adequate treatment, is feasible, aiming to minimize the patient’s discomfort and the potential of the patient’s prolonged postoperative hospitalization, even in the intensive care unit.
